# Post-ablation prolongation of atrioventricular nodal refractory period is correlated with long-term success of cryoablation for atrioventricular nodal reentrant tachycardia in the case of the persistence of a residual jump

**DOI:** 10.1007/s10840-012-9680-7

**Published:** 2012-05-05

**Authors:** Joelci Tonet, Antonio De Sisti, Natalia Pardo Restrepo, Denis Raguin, Walid Amara, Manlio F. Márquez, Philip Aouate, Xavier Waintraub, Faouzi Touil, Francoise Hidden-Lucet

**Affiliations:** 1 Cardiology Institute, Rhythmology Unit, Pitié-Salpêtrière Hospital, 47-83, boulevard de l’Hôpital, 75651 Paris, France; 2 Universidad CES, Calle 10 A No. 22, Medellín, Colombia; 3 Rhythmology Unit, Clinique de l’Europe, Amiens, France; 4 Cardiology Department, le Raincy-Montfermeil Hospital, Montfermeil, France; 5 Departamento de Electrofisiología, Instituto Nacional de Cardiología Ignacio Chávez, Juan Badiano 1, Col. Sección XVI, 14080 Mexico City, Mexico; 6 Institut de Cardiologie, Unité de Rythmologie, Hôpital de La Pitié-Salpêtrière, 47-83, Boulevard de l’Hôpital, 75651 Paris, France

**Keywords:** Atrioventricular nodal reentry tachycardia, Cryoablation, Radiofrequency catheter ablation, Slow pathway, Atrial echo, Atrioventricular nodal effective refractory period

## Abstract

**Purpose:**

A residual slow pathway after successful cryoablation for atrioventricular nodal reentrant tachycardia (AVNRT) is correlated with a higher recurrence rate. We described determinants of recurrence in subjects with a residual jump.

**Methods:**

We analyzed the data of subjects with acute successful slow pathway cryoablation for AVNRT using a 6-mm-tip cryocatheter. Success was defined as AVNRT non-inducibility. Patients with no baseline elicitable jump, no inducible AVNRT, and transient first atrioventricular (AV) block at the last site were excluded.

**Results:**

From 371 patients who underwent cryoablation from May 2002 to March 2011, 303 fulfilled the entry criteria (mean age, 41 ± 16; 222 women). Baseline AV nodal effective refractory period (ERP) was 272 ± 57 ms, postprocedural 331 ± 64 (*P* < 0.001), and the mean of the difference (Δ ERP) 60 ± 41. At the end of the procedure, 64 patients (21 %) had a residual jump, of whom 22 with a single echo. At 12 months follow-up, the actuarial recurrence-free rate was 70.3 % in patients with a residual jump and 86 % in those without (*P* = 0.01). In patients with a jump, only Δ AV nodal ERP was correlated with recurrence (37 ± 41 vs. 68 ± 47 ms; *P* < 0.04) while a single echo was not. The actuarial rate of recurrence was 60.8 % in patients with a Δ AV nodal ERP ≤ 30 ms and 18.8 % in those with a Δ AV nodal ERP >30 ms (*P* < 0.01).

**Conclusions:**

Suppression of slow pathway conduction is the optimal endpoint for AVNRT cryoablation. A residual jump can be tolerated if AV nodal ERP postcryoablation is prolonged >30 ms.

## Introduction

Slow pathway ablation is the treatment of choice for atrioventricular nodal reentrant tachycardia (AVNRT). Cryoablation is effective and safe, but its widespread use seems to be limited by a slightly lower long-term clinical efficacy when compared to radiofrequency (RF) ablation [1]. After an RF procedure, residual conduction through the slow pathway associated with AVNRT non-inducibility does not seem to modify long-term results [2]. Contrarily, the persistence of a residual slow pathway after cryoablation has been correlated with a higher recurrence rate [3–6]. In the present study, we described determinants of recurrence in subjects with a residual jump after successful cryoablation for AVNRT. We focused on correlation of the increment of atrioventricular (AV) nodal effective refractory period (ERP) measured at the end of the procedure with the clinical outcome of these patients.

## Methods

Patients from three French centers (Pitié-Salpêtrière Hospital, Paris; Le Raincy-Montfermeil Hospital, Montfermeil; and Clinic of Europe, Amiens) with successful cryoablation procedures for AVNRT performed with a 6-mm-tip cryocatheter (Freezor *Xtra* CryoCath®) were included. All centers adhered to inclusion/exclusion criteria, procedural methods, and endpoints. All subjects gave informed consent for the study. Exclusion criteria were: lack of baseline elicitable jump, non-inducibility of AVNRT at baseline, previous ablation attempts, other associated arrhythmias, underlying heart disease, transient first-degree AV block at the last ablation site, and the need for beta blocking agents.

### Procedure

**Fig. 1:**
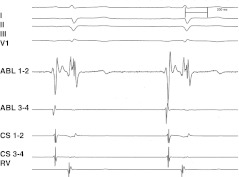
An example of optimal target site for AVNRT slow pathway cryoablation. Note an A/V ratio of about 1 and a stable nodal AV potential. *AVNRT* atrioventricular nodal reentry tachycardia, *A*/*V* atrioventricular

A standard electrophysiologic (EP) study was performed in the fasting state without sedation. Antiarrhythmic drugs were discontinued for at least five half-life periods. Dual AV nodal physiology was defined as a ≥50 ms increase in A2H2 in response to a 10 ms decrease during A1A2 stimulation [7]. If sustained tachycardia could not be induced, isoproterenol was administered. Slow pathway potentials in the Koch triangle were identified as the target site [7, 8] in a zone located anterior to the coronary sinus (CS), slightly below an ideal line delimiting the superior border of the CS ostium; an A/V ratio of ∼1 was generally preferred as a target (Fig. 1). Cryomapping was carried out first at a cryocatheter tip temperature of −30/40 °C for 30–45 s to test the cryo-effects on the target sites by using programmed stimulation. Cryoablation (−75/80 °C for 4/5 min) was initiated immediately following successful cryomapping, defined as abolition of the slow pathway or AVNRT noninducibility. If AVNRT remained inducible or AV block occurred, cryoablation was stopped and a further sequence of cryomapping and ablation at new target sites was repeated. A freezing–thaw–freezing cycle of cryoablation was not systemically carried out [1]. An EP study was continuously repeated during the waiting period of 30–45 min after the last cryoablation to check the procedure’s effectiveness, with and without isoproterenol infusion. Success was defined as non-inducibility of AVNRT. A residual slow pathway conduction, associated or not with a reproducible single echo, was permitted at the discretion of the senior operating electrophysiologist. Nodal refractoriness of the fast pathway was not systematically measured in this study. The final postprocedural AV nodal ERP was evaluated before isoproterenol administration. The cryoablation procedures were performed by 10 operators.

### Post-ablation management and follow-up

After cryoablation, no antiarrhythmic drugs were prescribed. During the follow-up, patients underwent trans-telephonic assessment of symptoms, rest ECG and 24-h Holter recording at 3 months and then every 6 months in our centers or by their referring physicians. Relapse was defined as the recurrence of index arrhythmia-typical symptoms of tachycardia with sudden onset and termination, tachycardia documented by ECG or 24-h Holter recording. The minimum observational period was set at 6 months.

## Statistical analysis

Continuous variables were expressed as mean ± SD. In the comparison between groups, ANOVA was used for continuous variables, and *X* ^2^ test for discrete variables. Correlation between long-term results in the follow-up and the different variables was performed using Cox’s regression model for univariate and multivariate analysis. Continuous variables were converted into dichotomic when appropriate. Following univariate analysis, factors with an associated *P* value < 0.10 were tested in multivariate analysis. A stepwise regression procedure was used to determine independent predictors. The *P* value for entry or removal of a variable from the regression model was 0.05 and 0.10, respectively. Actuarial graphs were constructed using the Kaplan–Meier method and differences were evaluated by log-rank test. A *P* value < 0.05 was considered significant. Statistical analysis was performed using SPSS 19 for Windows.

## Results

### Study group characteristics

Among 371 patients who had undergone AVNRT cryoablation from May 2002 to March 2011, 303 of them (mean age, 41 ± 16 years; 222 women) fulfilled the entry criteria (Table 1). Patients excluded were: 12 for initial unsuccessful procedures, 46 for absence of baseline jump or AVNRT inducibility, and 10 for transient first-degree AV block at the last effective ablation site. The contribution of each participating center to the study was: 301 patients from Pitié-Salpêtrière Hospital, 31 from Le Raincy-Montfermeil Hospital, and 30 from the Clinic of Europe.

**Table 1:** Clinical and procedural characteristics of the study population

Patients (*n*)	303 (%)
Type of AVNRT	
Slow-fast	284 (94)
Fast-slow	6 (2)
Slow-slow	13 (4)
AVNRT cycle length (ms)	331 ± 65
Cryoapplications (*n*)	6.3 ± 5.8
Total cryoapplication time (s)	1,052 ± 797
A/V at last effective site (ratio)	1 ± 0.65
Procedure time (min)	128 ± 47
Fluoroscopy time (min)	17 ± 13
Transient second/third degree AV block at last site (patients)	16
AV node ERP (ms)	
Baseline ERP	272 ± 57
Post-procedural ERP	331 ± 64
Δ ERP	60 ± 41
Post-procedural residual jump (patients)	64 (21)
Residual jump without single echo	42
Residual jump with echo	22
Recurrence (patients)	53 (17.5)
Redo procedure (patients)	24 (7.9)

*AA* antiarrhythmic, *AV* atrioventricular, *ERP* effective refractory period, *Δ ERP* difference between baseline and postprocedural ERP

Previous ineffective drugs in the 303 patients analyzed were 1.2 ± 1. Patients had the following AVNRT subtypes: slow-fast AVNRT in 284 patients, fast-slow in six, and slow-slow in the remaining 13. AVNRT cycle length was 331 ± 65 ms. The number of cryoapplications was 6.3 ± 5.8 per patient. Total cryoapplication time was 1,052 ± 797 s. The procedure and fluoroscopy times were 128 ± 47 and 17 ± 13 min, respectively. The A/V ratio at the last effective site was 1 ± 0.65. Transient second and third degree AV block at the last ablation site occurred in 16 patients (5.3 %). Baseline AV nodal ERP was 272 ± 57 ms, postprocedural ERP 331 ± 64 ms (*P* < 0.001), and the mean of the difference between baseline and postprocedural ERP (Δ ERP) 60 ± 41 ms. A postprocedural residual jump was elicited in 64 patients (21 %), of whom 22 with an associated single echo. During a mean follow-up of 45 ± 15 months, 53 (17.5 %) patients had recurrences. Redo procedures were performed in 24 patients (7.9 %).

### Predictors of recurrence in the study population

**Fig. 2:**
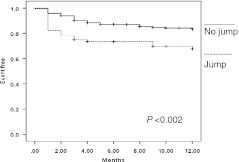
AVNRT recurrence-free event depending on postprocedural residual slow pathway. Δ AV nodal ERP: difference between baseline and post-procedural nodal effective refractory period

Recurrence was associated with a residual postprocedural jump (18/53 vs. 44/250 patients; *P* < 0.02), and a prolonged total cryoapplication time (1,389 ± 884 vs. 982 ± 762 s; *P* < 0.001; Table 2, Fig. 2). There was no significant difference concerning other clinical and procedural variables (including Δ AV nodal ERP) between patients with or without recurrence in the follow-up. At univariate and multivariate analysis, residual jump (univariate analysis HR, 2.05 [95 % CI 0.26–0.77], *P* < 0.02; multivariate analysis HR, 0.5 [95 % CI 1.03–3.8], *P* < 0.04), and total cryoapplication time >2,000 s (univariate analysis HR 2.42 [95 % CI 1.29–4.53], *P* < 0.01; multivariate analysis HR, 2.16 [95 % CI 1.14–4.08] *P* = 0.01) were significantly correlated with recurrence. At 12-months follow-up, the actuarial rate of recurrence-free patients was 70.3 % in the group with a residual jump (245 patients), and 86 % in those without a residual jump (*P* = 0.01).

**Table 2:** Procedural characteristics of patients with and without recurrence

	Total study population			Subgroup without residual jump			Subgroup with residual jump		
	Recurrence (*n* = 53)	No recurrence (*n* = 250)	*P*	Recurrence (*n* = 35)	No recurrence (*n* = 204)	*P*	Recurrence (*n* = 18)	No recurrence (*n* = 46)	*P*
Cryoapplication time (s)	1,389 ± 884	982 ± 762	<0.001	1,401 ± 855	962 ± 753	<0.005	1,384 ± 969	1,067 ± 881	NS
Residual jump	18 (34 %)	46 (18.4 %)	<0.02	–	–	–	–	–	–
Associated single echo (*n*)	8 (15 %)	14 (5.6 %)	<0.02	–	–	–	8 (44.4 %)	14 (30.4 %)	NS
Δ AV ERP (ms)	40 ± 5.8	42 ± 3	NS	66 ± 35	61 ± 40	NS	37 ± 41	68 ± 47	<0.04

*AV* atrioventricular, *ERP* effective refractory period, *Δ AV node ERP* difference between baseline and postprocedural AV nodal ERP

### Predictors of recurrence in subjects without a residual slow pathway at the end of the procedure

**Fig. 3:**
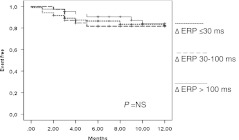
Patients without postprocedural residual slow pathway. AVNRT recurrence-free event depending on Δ AV nodal ERP. *AVNRT* atrioventricular nodal reentry tachycardia, *Δ AV nodal ERP* difference between baseline and postprocedural nodal effective refractory period

In the subgroup of patients without a residual jump at the end of the procedure, total cryoapplication time was more prolonged in patients with recurrence (1401 ± 855 vs. 962 ± 753 s; *P* < 0.005; HR: 3.14, 95 % CI: 1.49–6.61; *P* < 0.005; Table 2). No significant difference was found in terms of Δ AV nodal ERP between patients with and without recurrence (66 ± 35 vs. 61 ± 40; *P* = NS), whatever the cutoff value used (Fig. 3).

### Predictors of recurrence in patients with a residual slow pathway at the end of the procedure

**Fig. 4:**
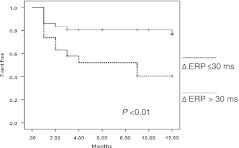
Patients with post-procedural residual slow pathway. AVNRT recurrence-free event depending on Δ AV nodal ERP. *AVNRT* atrioventricular nodal reentry tachycardia, *Δ AV* nodal *ERP* difference between baseline and post-procedural nodal effective refractory period

In patients with a residual jump, only Δ AV nodal ERP ms was significantly shorter in patients with recurrence (37 ± 41 vs. 68 ± 47 ms; *P* < 0.04; Table 3, Fig. 4). There was no significant difference in patients with and without recurrence in terms of total cryoapplication time (1,384 ± 969 vs. 1,067 ± 881 s; *P* = NS) and the presence of a single echo (8/18 vs. 14/46 patients; *P* = NS). At 12-months follow-up, the actuarial rate of recurrence-free patients was 62.9 % in the group with an associated single echo and 74.1 % in those without (*P* = NS).

**Table 3:** Predictive factors of recurrence in the study population (303 patients)

	Univariate analysis			Multivariate analysis		
Variables	HR	95 % CI	p	HR	95 % CI	*P*
Cryoapplication time >2,000 s (patients)	2.42	1.29–4.53	<0.01	2.16	1.14–4.08	=0.01
Residual jump (patients)	2.05	0.26–0.77	<0.02	0.5	1.03–3.28	<0.04

Using Cox’s model, Δ AV nodal ERP ≤30 ms was the best predictor of recurrence (HR: 0.25, CI: 0.09–0.69; *P* < 0.01). At 12-months follow-up, the actuarial rate of recurrence was 60.8 % in the group with AV nodal ERP ≤ 30 ms and 18.8 % in those with AV nodal ERP >30 ms (*P* < 0.01).

## Discussion

This study confirms that slow pathway persistence after cryoablation for AVNRT is correlated with a worse long-term outcome [1]. Also, as previously described [6], the persistence of a single echo in patients with a residual jump did not represent an adjunctive risk. A new finding was that the modification of slow pathway properties, specifically the prolongation of AV nodal ERP post-cryoablation, was associated with a better outcome in patients with residual slow pathway conduction. Contrarily, in those with complete slow pathway suppression at the end of the procedure, AV nodal ERP prolongation was not correlated with outcome.

### Global results for AVNRT cryoablation

Cryoablation for AVNRT has been the object of numerous studies in the last decade. In a recent review of the literature, we showed [1] in data pooled from 20 published studies including 2,351 patients [3–5, 9–25] that the initial procedural success rate for AVNRT cryoablation was 95 % (range, 85–99 %), not so far from the acute success described in RF series [26, 27]. Overall, the mean recurrence rate was 11 % (range, 7–19.7 %), higher than for RF catheter ablation, in which the recurrence rate has been reported as being between 3 and 5 % [26, 27].

### Residual slow pathway in patients treated by radiofrequency ablation

With RF energy, the persistence of a slow pathway with no more than a single echo can be an acceptable end-point. Recently, Stern et al. [2], in a meta-analysis including 1,204 patients from 10 studies, found that if isoproterenol was systematically used at the end of an RF procedure, no significant difference in recurrence rates could be found between patients with complete slow pathway suppression and those with a residual jump associated or not with a single echo. However, in studies in which isoproterenol was not systematically used, slow pathway suppression was associated with a lower risk of recurrence compared to slow pathway modifications.

### Residual slow pathway in patients treated by cryoablation

Globally, in studies including patients treated with cryoenergy, isoproterenol was frequently used at the end of the procedure, systematically or limited to patients with a residual slow pathway [1]. In the series of Gupta et al. [3], a single echo was induced in 20/71 (28 %) patients in the RF group and 19/71 (27 %) patients in the cryoablation group. Of these, one (5 %) and seven (36.8 %) patients, respectively, had documented arrhythmia recurrence (*p* < 0.05). Of note, in this study, isoproterenol was not uniformly used after ablation. In another study of subjects treated using only cryoenergy, Sandilands et al. [5] found that complete slow pathway conduction was associated with better long-term results. The procedure was considered successful if slow pathway suppression was achieved and/or in the presence of a single echo with AVNRT non-inducibility on isoproterenol. Recurrence rates were greater when slow pathway suppression was not achieved (8/12 patients, 66.7 %), compared with complete slow pathway conduction (11/129 patients, 8.5 %, *P* < 0.0001). Recurrence was significantly more likely if echo beats were still present after cryoablation: 12/130 (9.2 %) patients with no recurrence vs. 7/19 (36.8 %) patients with recurrence (*P* < 0.0001). Other authors found no significant differences in terms of recurrence in the case of the persistence of dual AV nodal physiology [4].

In the present extended study conducted in a selected population of patients with baseline elicitable jump and inducible AVNRT, the persistence of a residual slow pathway was associated with a higher incidence of recurrence, but the presence of an associated single echo did not modify results. These results confirm those from our recent report [6]. Also, a longer total cryoapplication time and a residual jump both independently characterized patients with recurrence.

### Slow pathway modifications in patients treated by cryoablation

There are scant data concerning slow pathway modification effects and the outcome in patients treated with cryoenergy [28]. In the present study, in the subgroup of patients without residual slow pathway conduction, the Δ AV ERP was not predictive of long-term results, whatever the cutoff used. The only factor correlated with recurrence was a prolonged cryoapplication time, which in this case may be considered as a marker of a difficult procedure, possibly linked to anatomical variants. Contrarily, in patients with slow pathway conduction at the end of the procedure, the ERP modification was correlated with recurrence. In the present study, a small variation of Δ AV ERP ≤ 30 ms appeared predictive of a worse outcome. Cryoapplication time was globally prolonged in this subgroup, but it was not correlated with recurrence.

Recently, Posan et al. [29] showed that in patients with persistent slow pathway conduction after RF ablation, but no recurrent AVNRT, slow pathway ERP increased, while the difference between the fast and slow pathway ERP was reduced, thus decreasing the induction window of reentry [30]. The same operating mechanism could be extrapolated to cryoablation. In the present study, we did not systematically evaluate fast pathway ERP, and in order to avoid confounding factors, we excluded from analysis patients with unwanted transient first-degree degree AV block after cryoablation suggesting fast pathway damage.

AV nodal ERP can of course fluctuate during the procedure, depending on patient stress, vagal reactions, and the residual effect of given drugs. Therefore, we evaluated the AV nodal ERP at baseline and before isoproterenol infusion at the end of the procedure. A cutoff of 30 ms appeared the best predictor at Cox’s model analysis.

### Clinical implications

Based on the data presented here, slow pathway suppression must be the endpoint of cryoablation of AVNRT. Slow pathway persistence (“residual jump”) can be tolerated if nodal ERP postcryoablation is prolonged more than 30 ms, without AVNRT inducibility with and without isoproterenol.

### Study limitations

In the present study, the recurrence rate was relatively high, probably linked to a long follow-up duration, a learning curve effect, the high number of operators with different levels of skill and training, and the fact that the main institution involved is a tertiary center where more difficult cases are generally addressed. Of note, the redo procedure rate was 7.9 %, strongly indicating that patients with recurrence who did not need further cryoablation had a substantial real-life improvement. Additionally, cryoablation of the slow pathway is a relatively new approach of catheter ablation for AVRNT compared to RF, and strategies to improve long-term results have been evaluated only in the recent past.

A parameter not evaluated in the present study was the “time to effect” during cryoablation, a phenomenon well known in RF ablation of accessory pathways, and also described in accessory pathway cryoablation in children [31].

## Conclusions

Suppression of slow pathway conduction is the optimal endpoint for AVNRT cryoablation. In patients with a residual jump, an associated single echo did not modify results, while a small prolongation in post-cryo ERP <30 ms was associated with a higher recurrence rate. Therefore, slow pathway persistence can be tolerated if nodal ERP is prolonged at the end of the procedure.
